# Early detection and differential serodiagnosis of *Mycoplasma hyorhinis* and *Mycoplasma hyosynoviae* infections under experimental conditions

**DOI:** 10.1371/journal.pone.0223459

**Published:** 2019-10-07

**Authors:** Luis G. Giménez-Lirola, Henrique Meiroz-De-Souza-Almeida, Ronaldo L. Magtoto, Aric J. McDaniel, Maria M. Merodio, Franco S. Matias Ferreyra, Korakrit Poonsuk, Igor R. H. Gatto, David H. Baum, Richard F. Ross, Paulo H. E. Arruda, Kent J. Schwartz, Jeffrey J. Zimmerman, Rachel J. Derscheid, Bailey L. Arruda

**Affiliations:** 1 College of Veterinary Medicine, Iowa State University, Ames, IA, United States of America; 2 São Paulo State University (Unesp), School of Agricultural and Veterinarian Sciences, Jaboticabal, SP, Brazil; University of Lincoln, UNITED KINGDOM

## Abstract

*Mycoplasma hyorhinis* (MHR) and *Mycoplasma hyosynoviae* (MHS) are common opportunistic pathogens in the upper respiratory tract and tonsils of swine. The identification of the specific species involved in clinical cases using conventional diagnostic methods is challenging. Therefore, a recombinant chimeric polypeptide based on the seven known variable lipoproteins (A-G) specific of MHR and a cocktail of surface proteins detergent-extracted from MHS cultures were generated and their suitability as antemortem biomarkers for serodiagnosis of MHR- and MHS-infection were evaluated by ELISA. *M*. *hyorhinis* and MHS ELISA performance, evaluated using serum samples collected over a 56-day observation period from pigs inoculated with MHR, MHS, *M*. *hyopneumoniae*, *M*. *flocculare*, or Friis medium, varied by assay, targeted antibody isotype, and cutoffs. The progressions of MHR and MHS clinical diseases were evaluated in relation to the kinetics of the isotype-specific antibody response in serum and bacterial shedding in oral fluids during the observation period. In pigs inoculated with MHR, bacterial DNA was detected in one or more of the 5 pens at all sampling points throughout the study, IgA was first detected at DPI 7, one week before the first clinical signs, with both IgA and IgG detected in all samples collected after DPI 14. The peak of MHS shedding (DPI 8) coincided with the onset of the clinical signs, with both IgA and IgG detected in all serum samples collected ≥ DPI 14. This study demonstrated, under experimental conditions, that both ELISAs were suitable for early detection of specific antibodies against MHR or MHS. The diagnostic performance of the MHR and MHS ELISAs varied depending on the selected cutoff and the antibody isotype evaluated. The high diagnostic and analytical specificity of the ELISAs was particularly remarkable. This study also provides insights into the infection dynamics of MHR-associated disease and MHS-associated arthritis not previously described.

## Introduction

*Mycoplasma hyorhinis* (MHR) [1] and *Mycoplasma hyosynoviae* (MHS) [2] are bacteria lacking cell walls within the class *Mollicutes*. Members of this class include several mycoplasmas that can be detected in various host species which differ significantly in their structure, habitat and growth requirements and are recognized as the smallest prokaryotic cells (0.2 and 0.8μm) capable of self-replication [3,4]. Although MHR and MHS are considered commensal microorganisms (i.e., detected by PCR in absence of clinical disease) of the upper respiratory tract and tonsils of swine, they can cause disease under specific conditions through mechanisms that are poorly understood. *M*. *hyosynoviae* has an affinity for joints, causing synovitis and arthritis, most commonly in pigs greater than 10-weeks-of-age [5,6]. *M*. *hyosynoviae*-related disease may affect a few individual pigs or cause epidemics with 10 to 50% of pigs affected before reaching market weight [7]. *M*. *hyorhinis*-associated disease has recently emerged as an important contributor to mortality in nursery pigs and has become a major concern within the U.S. swine industry [8,9]. *M*. *hyorhinis* colonization has been detected in nearly every production system in which it is sought [10], with herd prevalence that varies with age and among production systems [9]. Unlike MHS, MHR causes polyserositis (inflammation of several serous membranes) as well as arthritis [11–13].

Both MHR and MHS have emerged as important contributors of arthritis and lameness in growing pigs [14,15]. Lameness directly impacts pig’s welfare and indirectly results in economic loss due to reduced growth rates, increased mortality or culling, and costs associated with treatment. Thus, while there is a long-term need for better understanding of the disease pathogenesis and the immunologic responses, there is an immediate need for antemortem diagnostics able to inform interventions intended to prevent mycoplasma-associated arthritis.

A tentative diagnosis of MHR- and/or MHS-associated disease often can be made based on history, clinical signs and typical gross and microscopic lesions but antemortem diagnostic tools are not currently available. The diagnosis should be confirmed by culture of the agent in specific media (i.e., Friis, complemented Difco) from affected tissues, including serous membranes, joints or synovial fluid; although, isolation of *Mycoplasma* species is laborious and time-consuming [4, 11, 16–19]. Isolation from common carriage sites such as lung or tonsil would not be confirmatory for disease diagnosis. The use of molecular techniques such as real-time PCR (qPCR) have significantly improved the detection and diagnosis of MHR- and MHS-associated disease [8, 20–25]. In addition, several antibody-based methods have been used for evaluating exposure to MHR [26–29] and MHS [30–34] and/or vaccine compliance at the herd level [33–34]. However, there are no standardized or commercial antibody-based detection methods currently available for either MHR or MHS. Moreover, potential serologic cross-reactivity between different swine *Mycoplasma* species has also been reported [35–36].

In this study, the diagnostic performance of two serum antibody ELISAs, one based on a MHR chimeric VlpA-G recombinant protein and a second based on a cocktail of surface proteins extracted from MHS cultures were assessed using serum samples collected from groups of pigs experimentally inoculated with MHR, MHS, *Mycoplasma hyopneumoniae* (MHP), and *Mycoplasma flocculare* (MFLOC), or bacteria-free culture media (i.e., Friis; negative control). The kinetics of MHR and MHS isotype-specific serum antibody responses (IgG and IgA), bacterial shedding in oral fluids, and the progression of MHR and MHS clinical signs were evaluated during the observation period.

## Materials and methods

### Experimental inoculation and sample collection

A panel of specimens was generated by specific inoculation of cesarean-derived, colostrum-deprived (CDCD) pigs with different swine mycoplasmas (MHR, MHS, MHP, and MFLOC). The animal study was conducted at the Iowa State University Livestock Infectious Disease BSL-2 Isolation Facility (ISU-LIDIF) under the approval of the Iowa State University Institutional Animal Care and Use Committee. All pigs were closely observed twice daily by investigators and staff while at the facility and observations recorded.

Fifty CDCD 8-week-old pigs (cross breed between Large White and Yorkshire; Struve Labs, Manning, IA, USA) were randomly allocated into five groups of treatment housed in separate rooms and acclimated for seven days prior inoculation. Each treatment group was housed in a separate room with 5 pens (2 pigs per pen) equipped with nipple drinkers. Animals were provided an antibiotic-free commercial diet twice a day. Prior to inoculation, pigs were determined to be Mycoplasma-negative on the basis of real time polymerase chain reaction (qPCR) and enzyme-linked immunosorbent assay (ELISA) testing described herein and performed on serum, oral fluid, or tonsil scraping (MHS group only) samples collected prior to inoculation. Mycoplasma strain provenance, inoculum preparation, and route/s of inoculation for each group are shown in Table 1 [33, 37–39].

**Table 1:** Mycoplasma strains, inoculum preparation, dose, and route of inoculation used during experimental inoculations.

			Inoculum		Inoculation	
Group (No pigs)	Strain ^a^	Passage/media ^b^	Concentration ^c^	Reference	Route ^d^	Vol (mL)
*M*. *hyorhinis* (10)	38983	3^rd^/Friis	3.2 × 10^8^ CFU/ml	[11,13]	Tonsillar swabbing	2
					Intraperitoneal	2
*M*. *hyosynoviae* (10)	34428	3^rd^/Difco + mucin + turkey serum	2.1 × 10^9^ CFU/ml	[2, 40]	Tonsillar swabbing	2
					Intranasal (0.5 mL/nostril)	1
					Intravascular (ear vein)	1
*M*. *hyopneumoniae* (10)	232	Lung inoculum/Friis	1.0 × 10^6^ CCU/ml	[41]	Intratracheal	1
*M*. *flocculare* (10)	27399	59^th^/Friis	1.0 × 10^5^ CCU/ml	[41]	Tonsillar swabbing	2
					Intranasal	1
					Intratracheal	1
Negative control (10)	Friis medium	-	-	-	Intranasal (0.5 mL/nostril)	1

^a^ *M*. *hyorhinis* strain 38983 was originally field-isolated from a 9-week-old pig presenting pleuritic. *M*. *hyosynoviae* strain 34428 was originally field-isolated from a 15-week-old pig with arthritis. *M*. *flocculare* strain 27399 was originally isolated from a porcine pneumonic lung. *M*. *hyopneumoniae* strain 11 was passaged repeatedly in disease-free pigs resulting in strain 232.

^b^ *M*. *hyopneumoniae* strain 232 infected lung tissue was homogenated, diluted 1:100 in Friis medium and used as inoculum. The inoculum was thoroughly screened (PCR and routine aerobic and anaerobic culture) for other known swine pathogens and found negative.

^c^ CFU/mL: colony-forming units per mL; CCU/mL: color-changing units per mL. The purity of original seeds and final inoculum were evaluated by qPCR and microscopy staining (×1000 magnification) to rule out bacterial contamination including other *Mycoplasma* spp.

^d^ Routes of inoculation references: *M*. *hyopneumoniae* and *M*. *flocculare* [38]; *M*. *hyorhinis* [37]; *M*. *hyosynoviae* [5]; tonsillar swabbing [39].

Blood samples (n = 700) were collected from all pigs individually on DPIs -3, 0, 3, 7, 10, 14, 17, 21, 24, 28, 35, 42, 49, and 56. Blood was drawn from the jugular vein or cranial vena cava using single-use serum separation tube. Samples were then centrifuged 1,500 × *g* for 15 minutes and serum samples were stored at -80°C until used. Pen oral fluid samples (1 rope/pen; 5 pens/group) were collected daily from each group between DPI 0 to 56 using cotton ropes as described elsewhere [42]. At 56 DPI, surviving pigs (n = 49) were humanely euthanized by penetrating captive bolt (Accles and Shelvoke, Ltd., Sutton Coldfield, UK) followed by exsanguination, and gross lesions were observed. Histopathology was performed in a subset (n = 5) of pigs to provide supporting evidence of gross findings.

### *Mycoplasma* DNA extraction and real-time PCRs

Pathogen-specific PCRs were used to confirm infection, monitor patterns of shedding over time, and/or document the absence of cross-contamination between groups (rooms) over the observation period (–3 to 56 DPI). All DNA extraction and PCR testing were performed at the Iowa State University Veterinary Diagnostic Laboratory according to specific standard operating procedures accredited by the American Association of Veterinary Laboratory Diagnosticians. Total nucleic acids in each biological sample were extracted using the MagMAXTM Pathogen RNA/DNA kit (Applied Biosystems, Life Technologies, Carlsbad, CA, USA). In brief, 100 μl of sample, 20 μl of nucleic acid binding beads, and 150 μl of lysis/binding solution in absolute isopropanol were mixed in a 96-well round bottom plate. DNA extraction was performed using an automated 96-well magnetic particle processor (Thermo Scientific Kingfisher Flex, Thermo Fisher Scientific, Pitsburgh, PA, USA), as per manufacturer’s instructions. *Mycoplasma* spp. (MHR, MHS, MHP, MFLOC) cultivated in growth culture medium (positive extraction control), and nucleic acid-free extraction reagents (negative extraction control) were extracted together with the biological samples.

Primers and procedures for MHR and MHS qPCRs have been described elsewhere [25] except that a different set of primers were used for MHR (MHR-F 5’-GCATGTTGAACGGGATGTAGCATT-3’; MHR-R 5’-TGAAGCTGTGAAGCTCCTTTCTATTACTC-3’). In brief, 2.5 μl extracted DNA, 0.1 μl forward primer (0.1 mM), 0.1 μl reverse primer (0.1 mM), 12.5 μl Qiagen® quantitect SYBR Green (Qiagen®, Hilden, Germany), and 9.8 μl Qiagen nuclease free water (Qiagen). The real-time PCR reaction was conducted on an ABI 7500 Fast instrument (Applied Biosystem®, Foster city, CA, USA) as follow: 1 cycle of 95°C for 15 minutes; 45 cycles of 94°C for 15 seconds and 61°C for 30 seconds; 95°C for 15 seconds, and 60°C for 1 minute. The results were analyzed using an automatic baseline setting. Quantification cycle (Cq) values < 44 and melting temperatures of 75.9 ± 1°C and 81.5 ± 1°C were considered positive for MHR and MHS, respectively.

Primers, probe, and procedure for MHP qPCR was performed as described elsewhere [43]. In brief, 5 μl extracted DNA, 0.8 μl extracted DNA Ambion 25X Primer Probe Mix (Thermo Fisher Scientific, Waltham, MA), 5 μl TaqMan® Fast Virus 1 (Thermo Fisher Scientific), and 9.2 μl nuclease free water (Life Technologies). Nuclease free water (5 μl) was substituted to extract DNA as negative amplification control in each plate. The real-time PCR reaction was conducted on an ABI 7500 Fast instrument (Applied Biosystem®, Foster city, CA) as follow: 50°C for 5 minutes, 95°C for 20 seconds, 40 cycles of 95°C for 0.03 seconds, and 60°C for 30 minutes. The results were analyzed using an automatic baseline setting, where Ct values < 37 were considered positive for MHP.

Primers, probe, and procedure for MFLOC gel-based PCR was performed as described elsewhere [44]. In brief, 5 μl extracted DNA, 1 μl forward primer (0.01 mM), 2 μl reverse primer (0.01 mM), 12.5 μl Master Amp E (Qiagen), 0.2 Taq DNA Polymerase (Qiagen), and 7.3 μl Qiagen nuclease free water (Qiagen). Like for MHP, 5 μl of nuclease free water was substituted for extracted DNA as negative amplification control in each plate. The PCR reactions were conducted on 2720 Thermal cycler (Applied Biosystem®, Foster city, CA) as follow: 95°C for 4 minutes, 35 cycles of 94°C for 30 seconds, 57°C for 1 minute, 72°C for 1 minute, and 72°C for 10 minutes. The PCR product was subjected to electrophoresis using a QiAxcel (Qiagen). Clinical samples resulted in PCR product of 754 bp were considered positive for MFLOC.

### *M*. *hyorhinis* chimeric VlpA-G recombinant protein-based ELISA

The MHR genes coding for the seven known variable surface lipoproteins (VlpA through VlpG) were *in vitro* synthesized (Shanghai Genery Biotech Co., Ltd., Shanghai, China) in tandem using GS linker space sequences and consistent with a previous report [45], with the addition of a 5’ terminal thioredoxin (TRX) tag followed by a 6x histidine (His) tag. After amplification by PCR, the amplicon (1,122-nt) was cloned into a pET32a(+) expression plasmid, confirmed by sequencing (Genewiz Inc., Suzhou, China), and subsequently used to transform the *Escherichia coli* BL21(DE3) host strain (Invitrogen, Carlsbad, CA, USA). The transformants were grown in Luria-Bertani (LB) medium (Invitrogen, Thermo Fisher Scientific, Grand Island, NY, USA) containing 100 g/ml of ampicillin at 37°C with shaking at 250 rpm. When an A600 value of 0.6 was reached, 1 mM IPTG (isopropyl-D-thiogalactopyranoside) was added to induce over-expression of chimeric VlpA-G, and cultures were grown for 3 hours at 37°C. Cells were harvested by centrifugation at 3,500 × *g* for 15 min, resuspended in lysis buffer (20mM Tris-HCl and 500mM NaCl, pH 8.0), and disrupted by ultrasonication (Vibra-Cell sonicator; Sonics & Materials, Newtown, CT, USA). The crude extracts were centrifuged at 15,000 g for 30 min at 4°C, and the soluble expression of the His tag-fused chimeric VlpA-G polypeptide (36.8. kDa) was confirmed by SDS-PAGE analysis. VlpA-G (374 aa) was purified from the soluble fraction by sequential use of Ni-chelating SFF affinity chromatography (GE Healthcare, Pittsburgh, PA, USA), HiTrap Phenyl High Performance (HP) (GE Healthcare) hydrophobic interaction chromatography, and HiTrap Q HP anion exchange chromatography (GE Healthcare), consecutively applied according to the manufacturer’s instructions. Protein elutions were dialyzed against phosphate-buffered saline (PBS, pH 8) at 4°C. Chimeric VlpA-G polypeptide had the predicted size (36.8 kDa), as determined by SDS-PAGE and Western blot. No cleaving products were detected.

Ninety six-well polystyrene ELISA plates (Nunc, Thermo Fisher Scientific, Agawam, MA, USA) were coated with 100 μl of chimeric VlpA-G recombinant protein (0.8 μg/ml) in PBS (pH 7.4) and incubated at 4°C for 16 h. Plates were washed 5 times with PBS-T (0.1% Tween 20), blocked with a 1% (w/v) bovine serum albumin (BSA; Jackson ImmunoResearch Inc., West Grove, PA, USA) solution, incubated at room temperature (RT; 20–23°C) for 2 h, dried at 37°C for 3 h, and stored at 4°C in a sealed bag with desiccant packs. Serum samples, including positive and negative internal controls tested in duplicate, were diluted 1:50, incubated at 37°C for 1 hour, and washed 5 times with PBS-T. Then, 100 μl of peroxidase (HRP)-conjugated goat anti-pig IgG (Fc) antibody (Bethyl Laboratories Inc., Montgomery, TX, USA) diluted 1:25,000 was added to each well, and the plates were incubated at 37°C for 1 h. After plates were washed 5 times with PBS-T, the reaction was visualized by adding 100 μl of tetramethylbenzidine-hydrogen peroxide (TMB, Surmodics IVD, Inc., Eden Prairie, MN, USA) substrate solution to each well, and at room temperature for 5 min. The reaction was terminated by adding 100 μl of stop solution (Surmodics IVD, Inc.) to each well. The absorbance was measured at 450 nm using an automated plate reader (Molecular Devices, Sunnyvale, CA, USA). Serum antibody responses were expressed as sample-to-positive (S/P) ratios.

### *M*. *hyosynoviae* and *M*. *floculare* Tween 20 extracted surface proteins-based ELISAs

A pure culture of MHS was grown in Difco^TM^ medium supplemented with mucin and turkey serum and incubated at 37°C, as described elsewhere [2,40]. Likewise, MFLOC culture was grown in Friis medium as previously described [41]. In each case, cells were harvested by centrifugation and washed three times with PBS (pH 7.4). Surface proteins were extracted by mixing the bacterial pellet with a 2% Tween 20 solution (Sigma-Aldrich, St. Louis, MO USA), incubating the mix at 37°C for 90 min, and centrifuging at 59,573 × *g* for 30 min to remove cell debris. The supernatant was collected, diluted 1:100 in PBS (pH 7.4), used as antigen to coat (2–3 μg/well) 96-well plates (Nunc, Thermo Fisher Scientific), which were subsequently incubated at 4°C for 16 hours. Plates were washed with PBS-T, blocked with 1% BSA-based blocking solution, dried, and stored at 4°C until use. To perform the indirect ELISA, plates were loaded with 100 μl of sera diluted 1:50 in new born calf serum-based sample diluent, incubated at 37°C for 1 hour, and washed 5 times with PBS-T. Then, 100 μl of HRP-conjugated goat anti-pig IgG (Fc) antibody (Bethyl Laboratories Inc.) diluted 1:17,000 in a fetal bovine serum-based conjugate stabilizer for MHS and 1:15,000 for MFLOC was added to each well and the plates incubated at 37°C for 1 hour. Then, the plates were washed 5 times with PBS-T and the reaction was visualized by adding 100 μl of TMB (Surmodics IVD, Inc.) solution to each well and incubated at RT for 5 min. The reaction was stopped by adding 100 μl of stop solution (Surmodics IVD, Inc.), and the absorbance of each well was measured at 450 nm in an ELISA plate reader (Molecular Devices). ELISA results were expressed as sample-to-positive (S/P) ratios.

### Oxoid *M*. *hyopneumoniae* ELISA

The Oxoid^TM^ MHP ELISA (K004321-9; Oxoid Limited, Hampshire, UK) is a blocking ELISA for the detection of MHP antibodies in porcine serum that is based on a highly specific monoclonal antibody against a conserved epitope of the MHP 74KDa protein. The test was performed and interpreted according to the manufacturer’s instruction. Samples with a mean OD value (OD-S) < 50% of the OD of the buffered control (OD-BC) were interpreted positives, samples with OD-S ≥ 65% of the OD-BC were positives, and samples with OD-S between 50–65% were interpreted as equivocal.

### Data analysis

Statistical analyses were performed using commercial statistical software (SAS® Version 9.4, SAS® Institute, Inc., Cary, NC). The Fisher Exact Test was used to evaluate qualitative differences (*p* < 0.05) in the S/P isotype-specific (IgG, IgA) antibody responses within inoculation groups compared to the negative control group by day post-inoculation. Receiver operating characteristic curve (ROC) analysis was performed to estimate the area under the curve (AUC), and the diagnostic sensitivity, diagnostic specificity, and analytical specificity, including 95% confidence intervals, at various cutoffs for MHR and MHS indirect ELISAs. Serum samples (n = 148) collected from each group (MHR, MHS, MHP, MFLOC) before inoculation (DPI -3 and 0) plus all samples collected from the negative control group throughout the study (DPI -3 to 56) were used to estimate diagnostic specificity. The overall diagnostic sensitivity of the MHR ELISA was evaluated on positive samples (n = 68) collected from MHR inoculated pigs between DPI 17 and 56. The overall diagnostic sensitivity of the MHS ELISA was evaluated on positive samples (n = 63) collected from MHS inoculated pigs between DPI 21 and 56. Selected S/P cutoff values for both MHR ELISA and MHS ELISA were used to determine time of detection and over-time detection through the observational period. The analytical specificity of both ELISAs were evaluated using samples (n = 320; MHR) (n = 318; MHS) collected between DPI 7 and 56 from animals inoculated with heterologous *Mycoplasma* species.

## Results

### Clinical signs during the course of the infection

In eight (8/10) MHR-inoculated pigs, lameness, joint swelling, rough hair coat, and loss of condition, were noted on DPIs 11 to 56. Two pigs (2/10), both housed in the same pen, never developed clinical signs. Among the clinically affected pens (4/5), rough hair coat, loss of condition, and mild front and hind limb lameness were observed in two pens by DPI 11. At DPI 13, lameness with or without joint swelling, rough hair coats, depression or reluctance to move, and loss of conditions were observed in at least one of the two pigs within each affected pen (4/5). Two animals within the same pen were euthanized at DPI 24 due to anorexia and inability to ambulate as a result of polyarthritis and polyserositis. Lameness, rough hair coat, joint swelling, and loss of condition in all affected pens remained relatively unchanged from initial observations through the end of the study (DPI 56).

Clinical signs consistent with MHS*-*arthritis were observed in seven (7/10) MHS-inoculated pigs from DPI 8 until DPI 28. Clinical signs were first observed on DPI 8 in two pigs in two different pens. Clinical signs included swollen hocks, leg stiffness, and reluctance to move or altered gait. At DPI 10, seven animals (7/10) in four pens (4/5) were clinically affected. Clinical signs were not observed in three pigs, one of which died during blood collection on DPI 10. No clinical signs were observed in any pigs from the MHP- and MFLOC-inoculated groups.

### Pathologic lesions at necropsy

**Fig 1:**
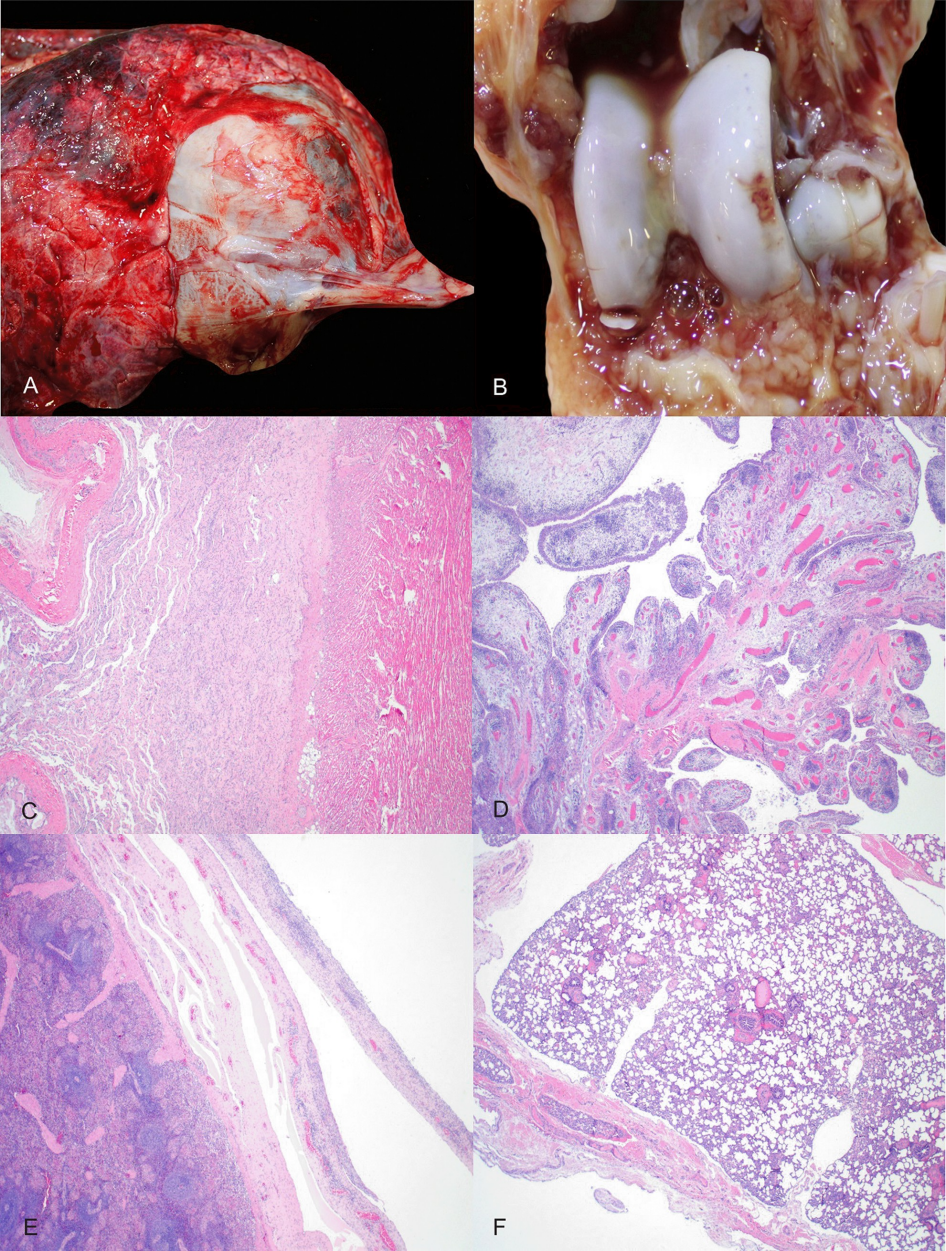
*Mycoplasma hyorhinis* pathologic lesions at necropsy. Animal ID 11, fibrosing epicarditis with marked thickening and opacity of the pericardium and attachment of the pericardium to the epicardium (A); Animal ID 17, Proliferative arthritis with hyperplasia of synovial villi (arrows), abundant serohemorrhagic joint fluid, and fibrin (arrowhead) (B); Animal ID 15, Fibrosing epicarditis, fibrous connective tissue is located between arrows (C); Animal ID 17, Proliferative synovitis with hyperplasia of synovial villi (arrows) dense multifocal inflammatory infiltrates expanding the subintima (arrowheads) (D); Animal ID 16, Fibrosing serositis with expansion of the serosal surface of the spleen by fibrous connective tissue (arrows) with entrapped multifocal inflammatory infiltrates (arrowheads) (E); Animal ID 20, Fibrosing pleuritis, fibrous connective tissue is located between arrows (F).

Fibrosing polyserositis lesions, i.e., fibrosing epicarditis, fibrosing pleuritis, fibrinous and fibrosing arthritis, and fibrosing peritonitis consistent with MHR infection were observed in nine (9/10) MHR-inoculated pigs at necropsy (Fig 1). Increased joint fluid volume was observed in three (3/10) MHS-inoculated pigs at necropsy. No lesions were observed in MHP- nor MFLOC-inoculated pigs at necropsy.

### Mycoplasma DNA detection in biological samples by qPCR

**Fig 2:**
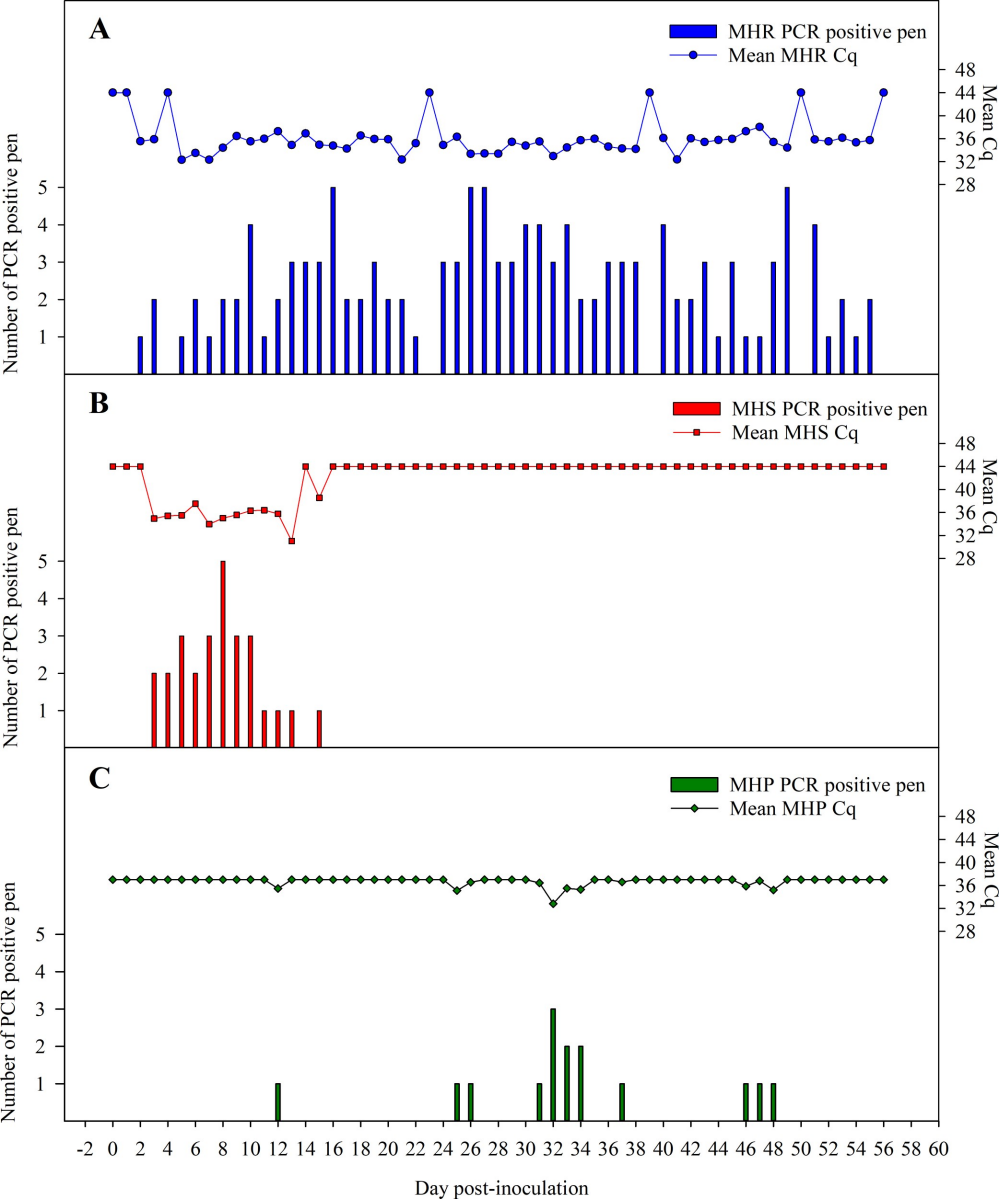
Detection of: A) *Mycoplasma hyorhinis* (MHR), B) *Mycoplasma hyosynoviae* (MHS), and C) *Mycoplasma hyopneumoniae* (MHP) shedding by qPCR in pen-based oral fluids collected independently from MHR-, MHS- and MHP-inoculated pigs over the course of 56 days. Results are presented as mean adjusted quantification cycle (Cq) (35 –sample Cq) of positive samples, and number of oral fluid PCR positive pens.

All samples collected from pigs in the different groups were tested PCR negative prior inoculation. Likewise, all animals in the control group were negative throughout the study. *M*. *hyorhinis* DNA was detected in one serum sample from one MHR-inoculated pig at DPI 10 (Cq 35.9). *M*. *hyorhinis* DNA was detected intermittently in pen oral fluids from DPI 2, ten days prior to the onset of clinical signs, through DPI 56 (Fig 2A).

*M*. *hyosynoviae* DNA was detected in two serum samples collected from two MHS-inoculated pigs at DPI 3 (Cq 33.3) and DPI 7 (Cq 34.4), respectively. *M*. *hyosynoviae* DNA was detected in one or more of the 5 pens from DPI 3, four days prior to the onset of the clinical signs, to DPI 15. Oral fluid samples from all MHS-pens (5/5) were MHS DNA-positive on DPI 8 (Fig 2B).

*M*. *hyopneumoniae* DNA was not detected in sera throughout the study. *M*. *hyopneumoniae* DNA was detected intermittently in oral fluid samples from 4/5 MHP-pens between DPI 12 to DPI 48 (Fig 2C). MFLOC DNA was not detected in serum nor oral fluid samples throughout the study.

### Mycoplasma serum antibody kinetics in response to infection

**Fig 3:**
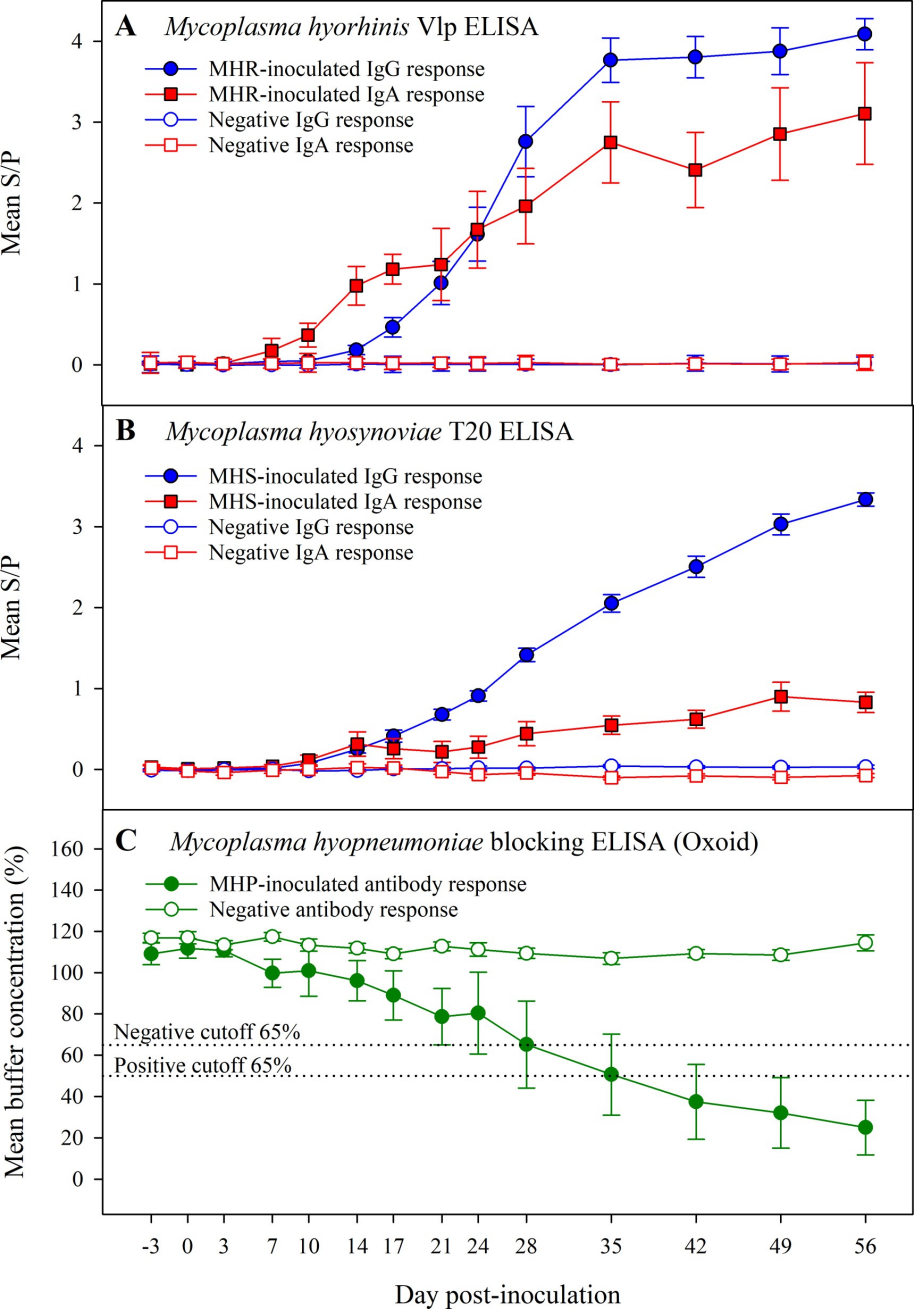
Kinetics of the antibody responses to: A) *Mycoplasma hyorhinis* (MHR), B) *Mycoplasma hyosynoviae* (MHS), and C) *Mycoplasma hyopneumoniae* (MHP) inoculated pigs (n = 10 pigs per inoculation group) over the course of 59 days and compared to the negative control group (n = 10 pigs). For MHR Vlp ELISA and MHS T20 ELISAs, results are presented as the mean (and standard error) of the sample-to-positive (S/P) ratios of the serum IgG (blue lines) or IgA (red lines) response over time. For the commercial MHP blocking ELISA (Oxoid), antibody response is presented as the mean (and standard error) of the buffer concentration (%) over time estimated following manufacturer’ instructions.

No serum antibody response against the Mycoplasma ELISAs was detected in serum samples collected prior to inoculation (DPI -3 and 0) or in samples collected from pigs in the negative control group throughout the study (Fig 3).

In the MHR-inoculated group (Fig 3A), a significant (*p* < 0.05) antibody response was first detected in MHR-inoculated pigs between DPI 10 (IgA) and DPI 14 (IgG) compared to pigs in the negative control group. The IgG and IgA antibody response to MHR increased with time post-exposure throughout the study. The MHR IgA antibody responses was detectable earlier than the IgG response. The IgA and IgG antibody response against MHR was significantly different on DPI 10 to 17 and 35 to 42 (*p* < 0.05).

Likewise, for MHS-inoculated pigs (Fig 3B), the first significant (*p* < 0.05) antibody response was detected between DPI 10 (IgG) to DPI 24 (IgA) compared to negative control group. Moreover, both MHS-specific IgG and IgA antibody responses increased thereafter until the end of the study. However, the IgG antibody response against MHS was significantly higher (*p* < 0.05) than the IgA response from DPI 21 to 56.

For MHP-inoculated pigs (Fig 3C), first seroconversion (positive result according to manufacturer’s cut-off) was detected between DPI 17 (1/10) and 24 (5/10). Antibody responses between MHP-inoculated and negative control were significantly different (*p* < 0.05) from DPI 35 to DPI 56. By DPI 49, 8/10 MHP-inoculated pigs seroconverted, while 2/10 pigs, located at different pens, did not show detectable antibody response during the study period. Contrary, for the MFLOC-inoculation group, no seroconversion was detected in any animal throughout the study.

### Diagnostic performance of *M*. *hyorhinis and M*. *hyosynoviae* ELISAs

**Fig 4:**
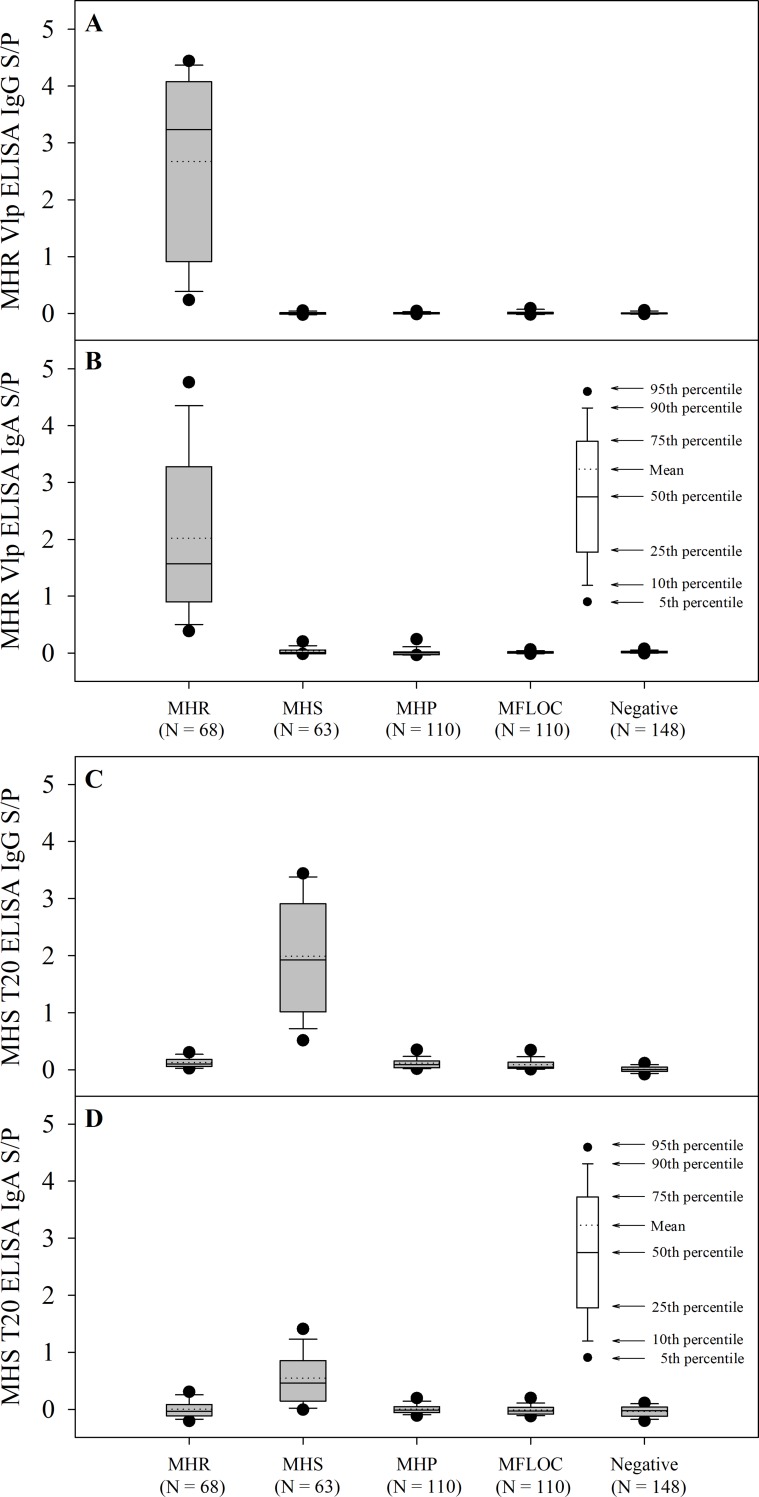
Distribution of *Mycoplasma hyorhinis* (MHR) Vlp ELISA and *Mycoplasma hyosynoviae* (MHS) T20 ELISA sample-to-positive (S/P) IgG (A, C) and IgA (B, D) responses in serum samples collected from pigs inoculated with MHR, MHS, *Mycoplasma hyopneumoniae* (MHP), *Mycoplasma flocculare* (MFLOC) or with culture medium (negative control).

The diagnostic performance of the MHR and MHS ELISAs were assessed by analyzing the distribution of the ELISA IgG and IgA S/P values in a subset of serum samples (n = 499) collected from the different treatment groups (Fig 4) using a ROC curve analyses. The AUC (%) estimated for MHR IgG ELISA was 0.998% (95% CI: 0.995, 1.000), 0.999 (95% CI: 0.998, 1.000) for MHR IgA ELISA and for MHS IgG and IgA ELISAs as 1.000 (95% CI: 0.995, 1.000) and 0.852 (95% CI: 0782, 0.910), respectively. The MHR ELISA showed excellent discrimination between positive and negative samples for both MHR IgG (Fig 4A) and IgA (Fig 4B). However, while the MHS ELISA showed a good discrimination for IgG (Fig 4C), the test discrimination capability for IgA was comparatively lower (Fig 4D).

The diagnostic sensitivity and specificity, and the analytical specificity of the IgG and IgA ELISAs associated to different S/P cutoff values for MHR ELISA and MHS ELISA are provided in Table 2 and Table 3, respectively.

**Table 2:** Diagnostic performance of the *Mycoplasma hyorhinis* isotype-specific Vlp ELISA based on experimental samples of known status.

Ig	Cutoff (S/P)	Diagnostic sensitivity (%) (95% CI)	Diagnostic specificity (%) (95% CI)	Analytical specificity (%) (95% CI)
IgG	0.1	98.4 (91.6, 100)	98.6 (96.0, 99.7)	97.5 (95.1, 98.9)
	0.2	98.4 (91.6, 100)	100 (98.3, 100)	99.4 (97.7, 99.9)
	0.3	93.8 (84.8, 98.3)	100 (98.3, 100)	99.4 (97.7, 99.9)
	0.4	90.6 (80.7, 96.5)	100 (98.3, 100)	99.7 (98.3, 100)
	0.5	87.5 (76.9, 94.5)	100 (98.3, 100)	100 (98.8, 100)
IgA	0.1	100 (94.4, 100)	98.2 (95.3, 99.5)	89.3 (85.4, 92.5)
	0.2	100 (94.4, 100)	100 (97.45, 100)	96.2 (93.5, 98.0)
	0.3	98.4 (91.6, 100)	100 (98.3, 100)	99.1 (97.3, 99.8)
	0.4	96.9 (89.2, 99.6)	100 (98.3, 100)	100 (98.9, 100)
	0.5	92.2 (82.7, 97.4)	100 (98.3, 100)	100 (98.9, 100)

**Table 3:** Diagnostic performance of the *Mycoplasma hyosynoviae* isotype-specific T20 ELISA based on experimental samples of known status.

Ig	Cutoff (S/P)	Diagnostic sensitivity (%) (95% CI)	Diagnostic specificity (%) (95% CI)	Analytical specificity (%) (95% CI)
IgG	0.1	100 (94.3, 100)	88.5 (83.3, 92.6)	55.2 (49.6, 60.8)
	0.2	100 (94.3, 100)	99.0 (96.4, 99.9)	82.5 (77.9, 86.6)
	0.4	100 (94.3, 100)	99.5 (97.3, 100)	97.1 (94.6, 98.7)
	0.5	96.8 (89.0, 99.6)	100 (98.2, 100)	99.7 (98.2, 100)
	0.6	93.7 (84.5, 98.2)	100 (98.2, 100)	100 (98.8, 100)
IgA	0.1	81.0 (69.1, 89.8)	88.5 (83.3, 92.6)	84.0 (79.5, 87.9)
	0.2	73.0 (60.1, 83.4)	98.5 (95.7, 99.7)	92.7 (89.2, 95.3)
	0.4	57.1 (44.1, 69.5)	100 (98.2, 100)	98.7 (96.8, 99.7)
	0.5	41.3 (29.0, 54.4)	100 (98.2, 100)	99.7 (98.2, 100)
	0.6	38.1 (26.2, 51.2)	100 (98.2, 100)	100 (98.8, 100)

**Fig 5:**
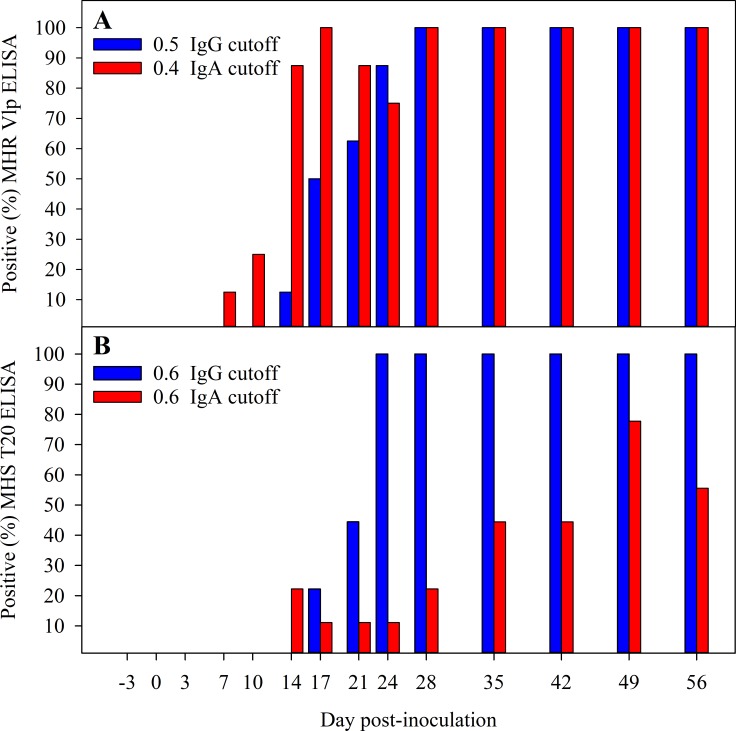
Proportion of ELISA positive serum IgG (blue bars) and IgA (red bars) samples over time following exposure to *Mycoplasma hyorhinis* (MHR (A)) or *Mycoplasma hyosynoviae* (MHS (B)). A S/P ≥ 0.50 cutoff for MHR Vlp IgG and 0.4 for MHR Vlp IgA ELISAs and S/P ≥ 0.6 for MHS T20 IgG and IgA ELISAs could be selected to ensure 100% diagnostic and analytical specificities.

Fig 5 shows the proportion of MHR and MHS ELISA positive samples over time post-exposure using selected optimal cut-offs. Fig 5A shows the proportion of positive serum IgG and IgA samples detected over time by the MHR ELISA using an S/P cutoff value of 0.5 for IgG and 0.4 for IgA, which provided 100% of diagnostic and analytical specificities. Likewise, Fig 5B shows the proportion of positive samples detected over time by the MHS ELISA using a cutoff of 0.6 for both IgG and IgA to ensure 100% diagnostic and analytical specificities.

## Discussion

In the last several years, there has been an increase in MHR-associated disease and MHS-associated arthritis in growing pigs resulting in significant production losses due to reduced growth rate, increased mortality or culling, and antibiotic costs [14,15]. Field diagnosis is based on clinical signs and gross lesions, a process which is complicated by the presence of other bacteria (e.g., *Haemophilus parasuis*, *Streptococcus suis*, and *Erysipelothrix rhusiopathiae*) causing fibrinous lesions comparable to those produced by MHR and MHS [20,46]. Conventionally, the diagnosis of MHR-associated disease and MHS-associated arthritis is achieved by demonstration of the agent from tissue showing typical lesions, including swabs collected from serosal surfaces, synovial fluid or fibrin from affected locations [4, 11, 16–17, 19]. Although highly diagnostically specific, the detection and identification of these agents by conventional methods can be difficult and time-consuming.

Despite the common occurrence and production losses attributed to MHR-associated disease and MHS-associated arthritis, the availability of well-characterized differential serologic assays and the implications that may exist between PCR results from oral fluids and clinical signs is limited. Accordingly, the primary goal of this study was to improve antemortem tools for early detection of MHR and MHS infections.

The suitability of a chimeric polypeptide based on a family of variable surface lipoproteins (Vlp) specific to MHR, and a cocktail of surface proteins extracted from MHS cultures as antemortem biomarkers for serodiagnosis of MHR-associated disease and MHS-associated arthritis were evaluated on serum samples in an ELISA platform.

The MHR Vlp family is composed of seven known members (i.e., VlpA, VlpB, VlpC, VlpD, VlpE, VlpF, and VlpG) with a continuous hydrophilic sequence projected externally from the bacteria membrane, and containing critical regions subject to immune recognition [44–47]. The Vlp system, unique to MHR, provides MHR with a mutational strategy based on high frequency phase variation of different *vlp* genes in expression and size by intragenic recombination. This system generates antigenic diversity among strains, which may be involved in cytoadhesion, evasion of the host immune response, and induction of chronic infections [47–51]. To overcome the divergence in the sequence among *vlp* genes and the variation in their expression, even lack of certain *vlp* genes, between MHR strains [52], a chimeric VlpA-G broad-spectrum MHR antigen derived from the seven known *vlp* genes ligated in-frame with an intervening linker was designed, cloned and overexpressed using an *E*. *coli* expression system which is easily scalable for high throughput antigen production [45].

Analyzing the kinetics of the antibody response to the MHR chimeric VlpA-G antigen, the early IgA response detected in serum by DPI 7, one week before the first clinical signs, was found particularly remarkable. The question of whether this IgA response might allow for detection of subclinical infections in presence of maternal IgG antibodies, as demonstrated for PRRSV [53], would need to be further investigated. The IgG response appeared later, between DPI 14–17, concomitantly with the progression of the clinical signs. Both IgA and IgG VlpA-G responses increased throughout the study, and their duration needs to be further investigated under experimental and field conditions against different MHR strains.

Unlike MHR, there is a lack of information regarding specific biomarkers of infection for MHS, which justified the use of a cocktail of MHS surface proteins as coating antigen for ELISA development. Typically, albeit with variations in the methodology for bacterial culture and antigen preparation, MHS surface proteins have been widely used for ELISA development [32–33, 36–37]. Previously, Gomes Neto *et al*. (2015) used an ELISA based on surface proteins extracted from MHS cultures following the same procedure described herein [25]. While they reported late seroconversion (DPI 35–49) and low overall detection rate (2/4 pigs) in a pilot-size animal study, the MHS ELISA described herein was capable of detecting antibodies earlier, between 14 to 17 DPI, approximately one week after the appearance of the first clinical signs and concomitantly with the cessation of the bacterial shedding in oral fluids. Although based on the same antigen, the differences in test performance could be due to differences in the set of experimental samples used for test evaluation, and differences on assay design/development, including test procedure and composition of ELISA reagents. Unlike the antibody kinetics described for MHR, the IgG response to MHS was significantly higher over time, and provided better diagnostic performance than the IgA response. The kinetics of the isotype-specific antibody response depends, among other factors, on the type of infection, specimens tested, antigen-specific response, and the intrinsic analytical sensitivity of the test. The biologic importance of the IgA response in MHS-associated disease and MHS colonization as well as the possibility of improving the detection of IgA generated in response to MHS, e.g., when more specific MHS antigen targets become available, warrants further investigation.

The overall diagnostic performance of the MHR and the MHS IgG/IgA ELISAs varied depending on the selected cutoff and the antibody isotype evaluated (Tables 2 and 3). A S/P ≥ 0.50 cutoff for MHR Vlp IgG and 0.4 for MHR IgA ELISAs and S/P ≥ 0.6 for MHS IgG and IgA ELISAs could be selected to ensure 100% diagnostic and analytical specificities while providing high detection rate over time, with 100% seropositive animals detected for both ELISAs after 24–28 DPI throughout the study. The high analytical specificity of the MHR and MHS ELISAs, evaluated on serum samples collected from pigs inoculated with heterologous *Mycoplasma* species, was particularly remarkable. The absence of serologic cross-reactivity between MHR-, MHS-, and MHP-inoculated pigs was demonstrated. However, 2/10 pigs in the MHP inoculation group did not show detectable antibody response during the study period. All pigs used in this study were from the same source (farm, genetic, age group) and all receive the same inoculum, provided by the same team and at the same time. The probability of infection by dose has not been established. Thus, at any specific dose, some proportion of animals may become infected; while others receiving the same dose will not. The spectrum of biological response (normal variation) and the role of the spectrum of host responses is a phenomenon which is well-recognized in the infectious disease literature [54].

Another limitation of this study was the lack of evidence of infection (shedding and/or seroconversion) in pigs from the MFLOC-inoculated group. In a previous study, Strasser et al. (1992) reported only a weak antibody response 6–8 weeks post-inoculation [38]. Previous studies demonstrated absence of clinical disease or lung lesions in response to single inoculation with *M*. *flocculare*, suggesting lack of infection likely due to poor colonization of the respiratory track [55, 56]. The possibility that antibody was not induced because of unsuccessful MFLOC infection could not be excluded; therefore, the serologic cross-reactivity of the MHR and MHS ELISAs against MFLOC seropositive animals warrants further investigation.

Previous reports suggested that oral fluids might represent a good alternative to individual animal sampling for monitoring MHR and MHS circulation/colonization in swine populations [8, 25]. However, our understanding of the implications that may exist between PCR results from oral fluids and clinical signs was limited due to the lack of well-established infection models for both MHR and MHS. This study describes the successfully development of a CDCD pig model to reproduce swine mycoplasmal arthritis in growing animals under experimental conditions. Most of the inoculated pigs developed clinical signs and pathological lesions consistent with MHR or MHS, respectively. *M*. *hyorhinis* oral fluid shedding was detected at high Cq values (Cq > 32) across pens throughout the study, suggesting that oral fluids could provide an easier specimen to determine herd status of colonization. The peak of MHS shedding (100%; DPI 8) coincided with the onset of the clinical signs; however, detection of MHS in oral fluids was limited compared to MHR being undetectable by DPI 14. Overall, the high Cq values obtained with both qPCRs indicated that the analytical sensitivity of current PCR protocols for MHR and MHS DNA detection in oral fluids could be further improved.

The identification of subclinically infected animals and the monitoring of disease progression in clinically affected swine herds is important for disease surveillance and management. Prior to this study, the detection of serum antibody against MHR or MHS has not been proven to correlate with the disease status of individual animals. This study demonstrated, under experimental conditions, that the combined use of two ELISAs based on a chimeric VlpA-G recombinant protein specific of MHR or a cocktail of surface proteins extracted from MHS cultures can be used for early detection, monitoring of infection, and differential serodiagnosis of MHR-associated disease and MHS-associated arthritis. Concurrent direct and indirect detection of MHR and MHS by PCR and ELISA, respectively, will provide insight into the dynamics of MHR and MHS infections in swine populations. Therefore, these antemortem detection tools could be used for intervention timing and evaluation of interventions to improve control of MHR-associated disease and MHS-associated arthritis. However, MHR and MHS DNA detection or isolation from common carriage sites such as lung or tonsil would not be confirmatory for MHR- and/or MHS-associated diseases. Therefore, PCR and serology results must be interpreted with caution.

To the authors’ knowledge, this is the first study to reproduce MHS-associated arthritis and MHR-associated disease in growing animals and monitor the clinical progression of MHR- and MHS-associated diseases in relation to the kinetics of the antibody responses in pig serum and bacterial shedding in pen oral fluids collected at different time points during the infection process. In doing so, this study provides not only MHR- and MHS-specific serologic assays but also insights into the infection dynamics of MHR-associated disease and MHS-associated arthritis not previously described.
